# Unveiling the Anti-Biofilm Property of Hydroxyapatite on *Pseudomonas aeruginosa*: Synthesis and Strategy

**DOI:** 10.3390/pharmaceutics15020463

**Published:** 2023-01-30

**Authors:** Davoodbasha MubarakAli, Kannappan Arunachalam, Murugan Lakshmanan, Bazigha Badar, Jung-Wan Kim, Sang-Yul Lee

**Affiliations:** 1 School of Life Sciences, B.S. Abdur Rahman Crescent Institute of Science and Technology, Chennai 620048, India; 2 Centre for Surface Coating and Technology, Department of Material Engineering, Korea Aerospace University, Goyang 10540, Republic of Korea; 3 Division of Bioengineering, Incheon National University, Incheon 22012, Republic of Korea; 4 State Key Laboratory of Microbial Metabolism, Department of Food Science and Technology, School of Agriculture and Biology, Shanghai Jiao Tong University, Shanghai 200240, China; 5 Department of Environmental Science, Amar Singh College, Cluster University Srinagar, Srinagar 190008, India

**Keywords:** hydroxyapatite, anti-biofilm, *Pseudomonas aeruginosa*, nanoparticles

## Abstract

Biofilm-related nosocomial infections may cause a wide range of life-threatening infections. In this regard, *Pseudomonas aeruginosa* biofilm is becoming a serious health burden due to its capability to develop resistance to natural and synthetic drugs. The utilization of nanoparticles that inhibit biofilm formation is one of the major strategies to control infections caused by biofilm-forming pathogens. Hydroxyapatite (HA) is a synthetic ceramic material having properties similar to natural bones. Herein, a co-precipitation method followed by microwave treatment was used to synthesize HA nanoparticles (HANPs). The resulting HANPs were characterized using X-ray diffraction and transmission electron microscopy. Then, their antibiofilm properties against *P. aeruginosa* ATCC 10145 were examined in vitro. The needle-shaped HANPs were 30 and 90 nm long in width and length, respectively. The synthesized HANPs inhibited the biofilm formation of *P. aeruginosa* ATCC 10145 in a concentration-dependent manner, which was validated by light and confocal laser scanning microscopy. Hence, this study demonstrated that HANPs could be used to control the biofilm-related infections of *P. aeruginosa*.

## 1. Introduction

Nanotechnology provides an excellent opportunity to explore the versatility of materials at the nanoscale compared to the bulk material. Nanoscale-level materials have shown superior properties in medicine, electronics, energy, and the environment. Many methods, such as physicochemical, biological, and blended methods, have been adopted to synthesize nanomaterials [1,2,3]. However, generally, there are limitations in the use of metallic, bimetallic, and composite materials in biological systems. Employing bio-mimetic nanoparticles allows the display of biocompatible and bioactive components, such as those based on biopolymer and hydroxyapatite [4,5].

Biofilms are multi-cellular, surface-attached microbial communities with distinct physiologic and architectural characteristics, which confer resistance to different classes of antibiotics [6]. Biofilm is a critical virulence factor for many bacterial pathogens that can cause chronic infections and are responsible for more than 80% of human microbial infections [7]. Most Gram-positive and Gram-negative bacteria can form biofilms, including *Escherichia coli*, *Klebsiella pneumoniae*, *Vibrio vulnificus*, *Enterococcus faecalis*, *Staphylococcus aureus*, *Streptococcus viridans*, *Proteus mirabilis*, and *Pseudomonas aeruginosa* [7,8].

The prevention of bacterial biofilm formation in nosocomial settings is very important. Biofilm-related nosocomial infections may cause a wide range of potentially fatal illnesses, threatening people in terms of health and cost [7,9]. Researchers worldwide have synthesized various kinds of metallic nanoparticles (NPs) and assessed their efficacy in treating bacterial infections, including those by antibiotic-resistant strains [3,10,11]. Most NPs made of silver, gold, copper, or zinc can inhibit biofilm formation [8,10,11,12]; however, these metallic NPs have exhibited cytotoxicity at elevated concentrations [13,14].

*P. aeruginosa* biofilm is becoming a serious issue due to its ability to develop resistance to natural and synthetic drugs [15]. Patients with cystic fibrosis are prone to biofilm-related infections by *P. aeruginosa* [15]. The use of NPs is a significant strategy to avoid biofilm-related infections. Antibiofilm applications of metallic NPs made of silver [16], copper [11], and other metals [5] have been practiced against bacterial and fungal infections. Still, the cost and toxicity of the metallic NPs are the major issues in utilizing them [17]. The chemical and biological similarities of hydroxyapatite (HA) to the natural components of bones and teeth make them suitable to exploit its potential as an alternative method for treating biofilm-related infection. In this regard, HANPs, either alone or in combination with other materials, has shown potential against various bacterial pathogens [5,18]. HANPs are also used as materials for implants and coating due to their potential to inhibit adverse inflammatory reactions and bacterial adherence [19,20]. Based on these postulations, this study is focused on the inhibition of the *P. aeruginosa* biofilm using HANPs at various concentrations.

## 2. Materials and Methods

### 2.1. Materials

Calcium hydroxide [Ca(OH)_2_], phosphoric acid (H_3_PO_4_), and Luria–Bertani (LB) medium were purchased from Himedia Co. (Mumbai, India), and the reagents used in this study (99.9% purity) were purchased from Sigma Aldrich (St. Louis, MO, USA).

### 2.2. Synthesis and Characterization of HANPs

**Figure 1:**
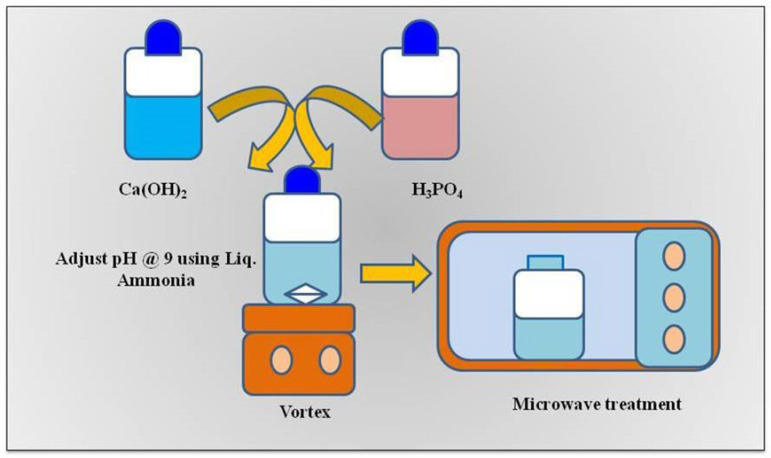
Schematic presentation illustrating the microwave-assisted synthesis of HANPs.

HANPs were synthesized using the microwave-assisted co-precipitation method reported previously [21]. Briefly, calcium hydroxide [Ca(OH)_2_] and phosphoric acid (H_3_PO_4_) were used as precursors for HANP preparation at a concentration of 1 mM and 90%, respectively. Each solution of H_3_PO_4_ and Ca(OH)_2_ was stirred for two hours. Then, the H_3_PO_4_ solution was added dropwise to the Ca(OH)_2_ solution while stirring. The solution mixture was left undisturbed for 2 h to obtain a precipitate. The precipitate was then washed with distilled water twice, and the pH of the precipitate was adjusted to 9 using liquid ammonia. The solution was dried for 10 min using a microwave oven (20PG3S, IFB, Kolkata, India) (Figure 1). The dried HANP powder was used for X-ray diffraction (XRD: SmartLAB, Rigaku Co., Tokyo, Japan) and transmission electron microscopy studies (TEM, JSM-JEOL, Tokyo, Japan).

### 2.3. Bacterial Strain and Culture Conditions

*Pseudomonas aeruginosa* ATCC 10145 was used in this study. The pathogen was cultured on Luria–Bertani agar plates (Bacto tryptone 0.5%, yeast extract 1.0%, NaCl 0.5%, agar 1.5%; pH 7.0) at 37 °C overnight and kept at 4 °C. For the biofilm assay, the bacteria were cultured for three hours overnight. Then, OD_600_ of the test broth was adjusted to 0.08 to 1.0 (McFarland standard, 1.5 × 10^8^ CFU mL^−1^) using the standard cell suspension (1 × 10^6^ CFU mL^−1^) and used for all biofilm-related experiments [22].

### 2.4. Antibiofilm Property of HANPs

#### 2.4.1. Static Biofilm Assay

The rate of biofilm inhibition was determined using a static biofilm assay. The biofilm of *P. aeruginosa* ATCC 10145 was treated with HANPs at various concentrations (10, 25, and 50 μg mL^−1^) in a 24-well polystyrene plate. Following the method reported previously [22], the standard cell suspension of *P. aeruginosa* ATCC 10145 was inoculated in 1 mL of LB broth containing various concentrations of HANPs and incubated at 37 °C for 24 h. After incubation, the planktonic cells from the wells were removed and thoroughly rinsed with distilled water twice. The biofilm was stained by adding an equal volume of crystal violet solution (0.4%, w v^−1^) to the wells and was left to stand for 10 min. Then, the dye was removed, and the wells were washed with distilled water to remove the excess stain. For the quantitative assay, an equal volume of absolute ethanol or 30% glacial acetic acid was added to dissolve the biofilm stained by crystal violet [23]. Then, the optical density of the solution was measured at 570 nm. The biofilm inhibition rate was calculated using Formula (1),

Biofilm inhibition (%) = ([Control OD_570_ − Test OD_570_]/Control OD_570_) × 100 (1)

#### 2.4.2. In Situ Analysis of Biofilm Formation

The effect of HANPs on the *P. aeruginosa* ATCC 10145 biofilm architecture according to the HANP concentrations was studied using a light microscope (Micros, Ansfelden, Austria) and a confocal laser scanning microscope (CLSM; LSM 710, Carl Zeiss, Jena, Germany). HANPs at various concentrations (10, 25, and 50 µg mL^−1^) were assessed for antibiofilm activity against *P. aeruginosa*. The pathogen was inoculated into a 24-well microtiter plate containing glass slides (1 × 1 cm) dipped in LB broth with and without HANPs. The pathogen was allowed to form the biofilm on the glass slides by incubating at 37 °C for 24 h. Following incubation, the glass slides were taken from the wells and washed with phosphate-buffered saline (PBS, pH 7.4) to remove the planktonic cells. For the light microscopic analysis, the glass slides were stained with crystal violet as described in Section 2.4.1, and the biofilm was observed under a light microscope at 400× (Micros, Austria) [24].

For CLSM analysis, the biofilm-embedded glass slides were stained using 0.1% acridine orange (w v^−1^) for 5 min in the dark. Then, they were washed with distilled water briefly and air-dried in the dark. Morphological changes in the biofilm architecture formed on the glass slides were observed and documented using a CLSM [25,26].

## 3. Results and Discussion

### 3.1. Synthesis and Characterization of HANPs

**Figure 2:**
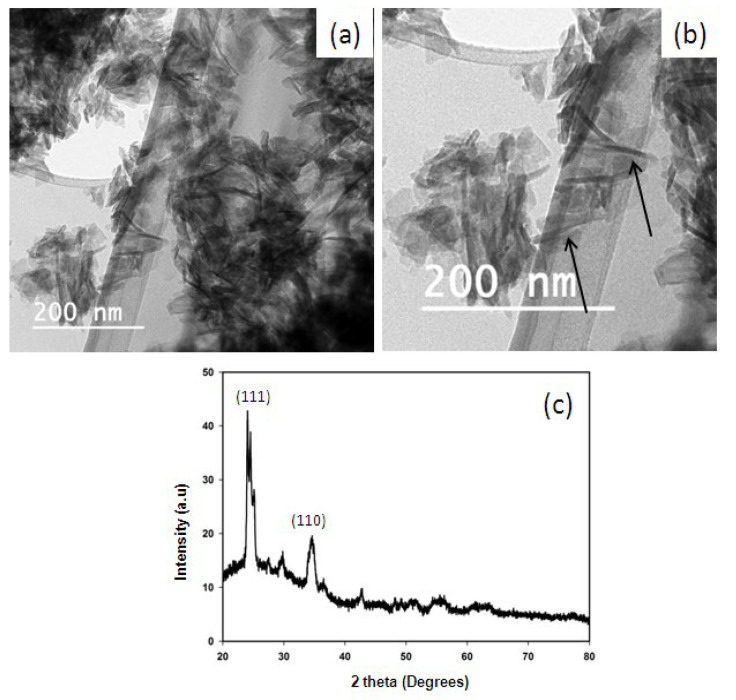
Characterization of HANPs prepared using the microwave method: HRTEM images showing the needle-shaped nanoparticles of 30 nm width and 90 nm length (**a**,**b**) and arrow indicate the needle shapted nanoparticles; XRD analysis of HANPs showing the crystalline nature of the particle with planes at (111) and (110) (**c**).

HANPs were synthesized using the microwave-assisted method, as illustrated in Figure 1, by observing precipitation formation. HR-TEM analysis revealed that the synthesized HANPs had a needle-like shape with an average of width of 30 nm and length of 90 (Figure 2a,b). Other studies have reported the synthesis of HANPs from stimulated body fluid and CaP Tris solutions using the precipitation method, wherein the HR-TEM images showed that average particle sizes of the HANPs synthesized from stimulated body fluid and CaP Tris solutions were 90–600 nm and 950–2000 nm, respectively [27]. The result of the XRD analysis of the synthesized HANPs is shown in Figure 2c. A strong diffraction peak was observed around 2θ values of 25 and 34, corresponding to the orientation plane (111) and (110), respectively. HA synthesized using *Klebsiella pneumoniae* that could solubilize phosphate showed a similar XRD pattern to that obtained in this study [21].

### 3.2. Quantitative Analysis of P. aeruginosa ATCC 10145 Treated with HANPs

**Figure 3:**
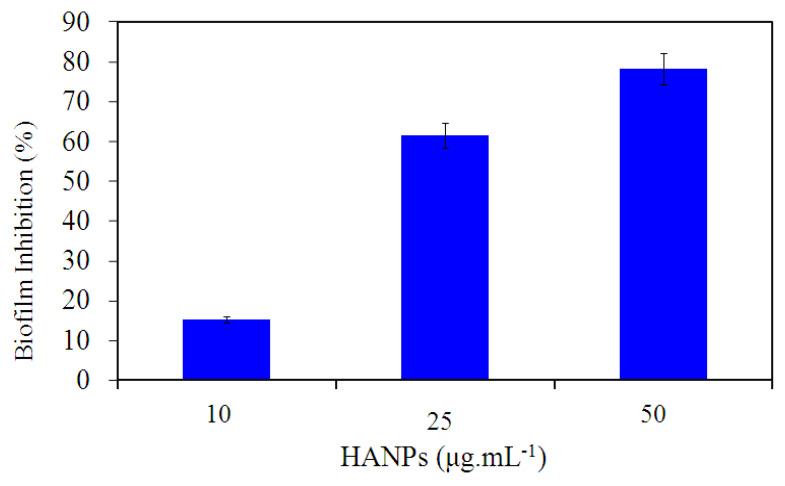
Antibiofilm property of HANPs against *P. aeruginosa* biofilm ATCC 10145 treated with various concentrations of HANPs for 24 h (*p* ≤ 0.05).

The capability of the HANPs to reduce the risk of *P. aeruginosa* ATCC 10145 attachment to the surface of the polystyrene microtiter plate was determined using the crystal violet staining method. The HANPs showed dose-dependent biofilm inhibitory activity against *P. aeruginosa* ATCC 10145. The HANPs showed a maximum of 78.2% antibiofilm activity against *P. aeruginosa* ATCC 10145 at a concentration of 50 μg mL^−1^ (Figure 3). In contrast to the negative control, which contains only HA at the higher concentration used in this study (data not shown), HANPs significantly reduced the biofilm formation in *P. aeruginosa* ATCC 10145. This result indicated that the HANPs could prevent the initial colonization of *P. aeruginosa* ATCC 10145 on the polyvinyl surface. Similarly, a recent study by Beyene and Ghosh [28] showed the antibiofilm properties of nanocomposite containing green synthesized zinc oxide and HANPs. Compared to the HANPs alone, the nanocomposite showed pronounced activity against the biofilm formation of *S. aureus* and *E. coli*. Likewise, another study also proved the antibiofilm activity of HANPs against *Staphylococcus aureus*, *Bacillus subtilis*, *K. pneumoniae*, *Candida albicans,* and *Escherichia coli* [29].

Similarly, recent work by Higuchi et al. (2022) reported that biomaterials containing HANPs and nano zinc oxide–silver sonicated on electrospun fibers of poly (D, L-lactic acid)/poly (lactic-co-glycolic acid) proliferated the growth of osteoblast cells [30]. Poly (D, L-lactic acid)/poly (lactic-co-glycolic acid) sonicated with nano zinc oxide–silver biomaterial inhibited bacterial growth with a high toxicity profile towards osteoblasts. The addition of HANPs to poly (D, L-lactic acid)/poly (lactic-co-glycolic acid) sonicated with nano zinc oxide–silver biomaterial decreased the toxicity towards osteoblasts. These results show the biocompatibility of HANPs towards osteoblast cells. However, the biomaterial containing HANPs and nano zinc oxide–silver did not exhibit antibacterial activity against *S. aureus* and *E. coli*. Similarly, in another study, samarium-doped hydroxyapatite coatings render antibiofilm activity without inhibiting the growth of *S. aureus* and *P. aeruginosa* [31]. Hence, these data suggest HANPs as a biocompatible material with antibiofilm potential against bacterial pathogens and necessitate a deeper understanding of the action mechanism of HANPs against the biofilm formation of bacterial pathogens, especially *P. aeruginosa*.

### 3.3. Qualitative Analysis of P. aeruginosa ATCC 10145 Treated with HANPs

**Figure 4:**
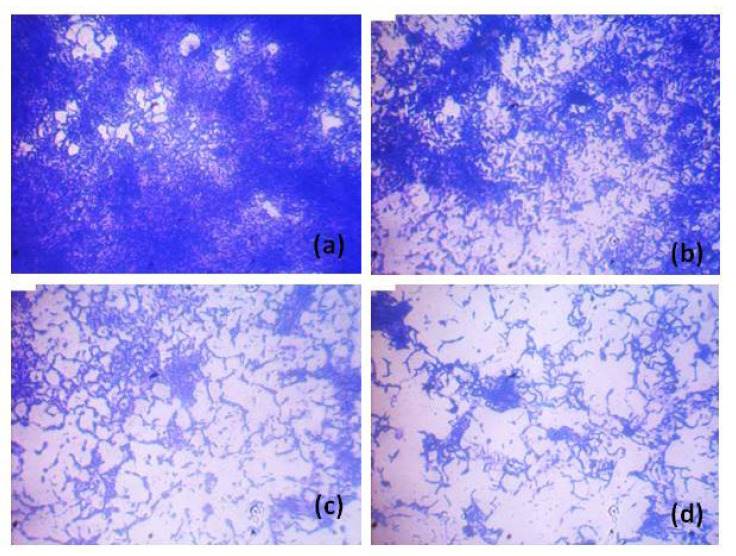
Light microscopy analysis showing the biofilm inhibitory potential of HANPs against *P. aeruginosa* ATCC 10145 at increasing concentrations. Control, treated with HANPs at 0 µg mL^−1^ (**a**); biofilm formed in the presence of HANPs at 10 µg mL^−1^ (**b**), 25 µg mL^−1^ (**c**), and 50 µg mL^−1^ (**d**).

**Figure 5:**
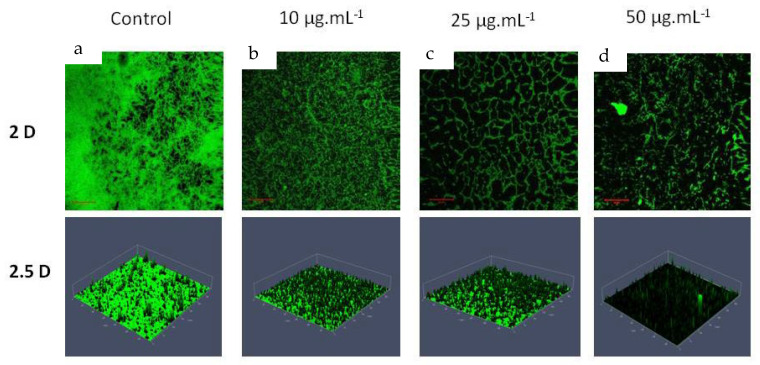
CLSM analysis showing the biofilm inhibitory potential of HANPs against *P. aeruginosa* ATCC 10145 at increasing concentrations. Control, treated with HANPs at 0 µg mL^−1^ (**a**); biofilm formed in the presence of HANPs at 10 µg mL^−1^ (**b**), 25 µg mL^−1^ (**c**), and 50 µg mL^−1^ (**d**).

Light microscopy analysis revealed the ability of HANPs to inhibit the biofilm of *P. aeruginosa* on the surface of the glass. The biofilm matrix formed in the presence of HANPs was more distorted than the control. The gradual and visible reduction in the biofilm matrix was observed with the increasing concentration of HANPs (Figure 4). CLSM is a sophisticated microscopy that provides a detailed analysis of biofilm morphology using 2D and 2.5 D detection. CLSM imaging revealed that HANP treatment changes the structural morphology of the *P. aeruginosa* biofilm, such as the biofilm density and multilayered biofilm formation represented by its height (Figure 5 (2.5D)). The result proved the potential of HANPs as surface modulators inhibiting the initial attachment of the *P. aeruginosa* cells, which might modulate the rigidity of biofilms and allow easy permeability of antimicrobial agents. Therefore, the microscopy analysis clearly stated that the change in the morphology of the biofilm was due to the biofilm inhibitory effect of HANPs.

*P. aeruginosa* biofilm remains a significant menace to people enduring acute diseases such as cystic fibrosis, diffuse panbronchiolitis, and chronic obstructive pulmonary disease [15]. Moreover, *P. aeruginosa* infection, especially biofilm formation, is frequent in immunocompromised patients and people with implants such as catheters and contact lenses [32]. Therefore, conventional treatment with antibiotics has become useless against the *P. aeruginosa* biofilm, and a higher concentration of antibiotics is required for effective treatment [33,34]. The use of NPs for treating biofilm-related infections has recently been on the rise. The present study elucidated HANP synthesis and its capability to inhibit *P. aeruginosa* biofilm formation.

HA is a bioceramic material with excellent biocompatibility, osteoconductivity, and bioactivity [28,35,36]. Clinically, HA possesses a wide range of medical applications, such as serving as a filling material for obstructing tooth cavities or bone fractures [37], and a coating material [38]. So far, the efficacy of HANPs against the biofilm of clinically relevant bacterial pathogens has been evidenced alone or in combination with other nanoparticles and antibiotics [13,28,29,39]. Hanning et al. [40] utilized HA microcrystals to prevent the accumulation of *Staphylococcus mutans* biofilm in vitro. The results obtained in this study are the first and foremost work revealing the capability of HANPs to inhibit the biofilm of Gram-negative bacteria *P. aeruginosa* via qualitative and quantitative analysis. Only a small number of investigations have been carried out to analyze the activity of HANPs against bacterial biofilm. Further, HA possesses more extended stability, revealing the potential of HA as an effective coating material over different surfaces [41]. Thus, further incorporating metallic NPs and antibiotics with the HANPs synthesized at 10 min might increase the biofilm inhibitory potential against *P. aeruginosa* and other pathogenic bacteria.

## 4. Conclusions

HANPs can effectively impede the biofilm formation of *P. aeruginosa* ATCC 10145. Clear evidence has shown that HANP treatment significantly reduced biofilm development and rendered apparent morphological changes in the biofilm architectures of *P. aeruginosa* ATCC 10145. We believe that the findings of this study will attract more researchers to exploit the application-oriented use of HANPs to combat biofilm-associated *P. aeruginosa* infections.

## Data Availability

Data Unavailable due to Privacy.
